# The expanding phenotype of hypokalemic periodic paralysis in a Japanese family with p.Val876Glu mutation in *CACNA1S*

**DOI:** 10.1002/mgg3.1175

**Published:** 2020-02-27

**Authors:** Mari Kurokawa, Michiko Torio, Kazuhiro Ohkubo, Vlad Tocan, Noriko Ohyama, Naoko Toda, Kanako Ishii, Kei Nishiyama, Yuichi Mushimoto, Ryuichi Sakamoto, Maki Nakaza, Riho Horie, Tomoya Kubota, Masanori P. Takahashi, Yasunari Sakai, Masatoshi Nomura, Shouichi Ohga

**Affiliations:** ^1^ Department of Pediatrics Graduate School of Medical Sciences Kyushu University Fukuoka Japan; ^2^ Department of Medicine and Bioregulatory Science Graduate School of Medical Sciences Kyushu University Fukuoka Japan; ^3^ Department of Functional Diagnostic Science Osaka University Graduate School of Medicine Osaka Japan; ^4^ Division of Endocrinology and Metabolism Department of Internal Medicine Kurume University School of Medicine Fukuoka Japan; ^5^ Present address: Department of Community and Emergency Medicine Ehime University Graduate School of Medicine Toon Japan

**Keywords:** *CACNA1S*, creatine kinase, hypokalemic periodic paralysis, insulin secretion

## Abstract

**Background:**

Hypokalemic periodic paralysis (HypoPP) is an autosomal dominant disease characterized by the episodic weakness of skeletal muscles and hypokalemia. More than half patients with HypoPP carry mutations in *CACNA1S*, encoding alpha‐1 subunit of calcium channel. Few reports have documented the non‐neuromuscular phenotypes of HypoPP.

**Methods:**

The proband is a Japanese woman who developed HypoPP at 6 years of age. An excessive insulin secretion with the oral glucose tolerance test rationalized that she had experienced frequent attacks of paralysis on high‐carbohydrate diets.

**Results:**

Voglibose and acetazolamide effectively controlled her paralytic episodes. Her 8‐year‐old son and 2‐year‐old daughter started showing the paralytic symptoms from 4 and 2 years of age, respectively. Laboratory tests revealed high concentrations of creatinine kinase in serum and elevated renin activities in plasma of these children. The targeted sequencing confirmed that these three patients had an identical heterozygous mutation (p.V876E) in *CACNA1S*.

**Conclusion:**

Our data indicate that the p.V876E mutation in *CACNA1S* contributes to the early onset of neuromuscular symptoms and unusual clinical phenotypes of HypoPP.

## Graphical abstract

Our study confirmed that the Japanese family in this report had a heterozygous mutation of p.V876E in CACNA1S. As the third family carrying the rare variant, these findings provide evidence that p.V876E in CACNA1S is linked to the early onset of neuromuscular symptoms and unusual clinical phenotypes of HypoPP.

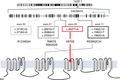

## 1 INTRODUCTION

Hypokalemic periodic paralysis (HypoPP) is an autosomal dominant disorder characterized by recurrent episodes of paroxysmal weakness in the skeletal muscle (Statland et al., 2018). The episodic paralysis lasts a few hours to days in association with low serum potassium, and the symptoms typically start in early teen years (Miller et al., 2004).

Among known genetic causes, mutations in the calcium channel gene *CACN1AS* (OMIM 114,208) account for 55%–70% of patients with HypoPP (Matthews et al., 2009; Miller et al., 2004). This gene encodes the voltage‐gated calcium channel alpha subunit, which plays an important role in Ca^2+^‐mediated excitation and contraction coupling. To date, nine missense mutations have been identified in patients with HypoPP. The p.Val876Glu mutation is associated with the earlier onset of recurrent paralysis than other mutations (Ke, Gomez, Mateus, Castano, & Wang, 2009; Yang, Zhang, & Xing, 2015). However, non‐neuromuscular phenotypes of this mutation have not been documented. We herein report an endocrine abnormality in a Japanese family carrying a p.Val876Glu mutation in *CACNA1S*.

## 2 CASE PRESENTATION

**Figure 1:**
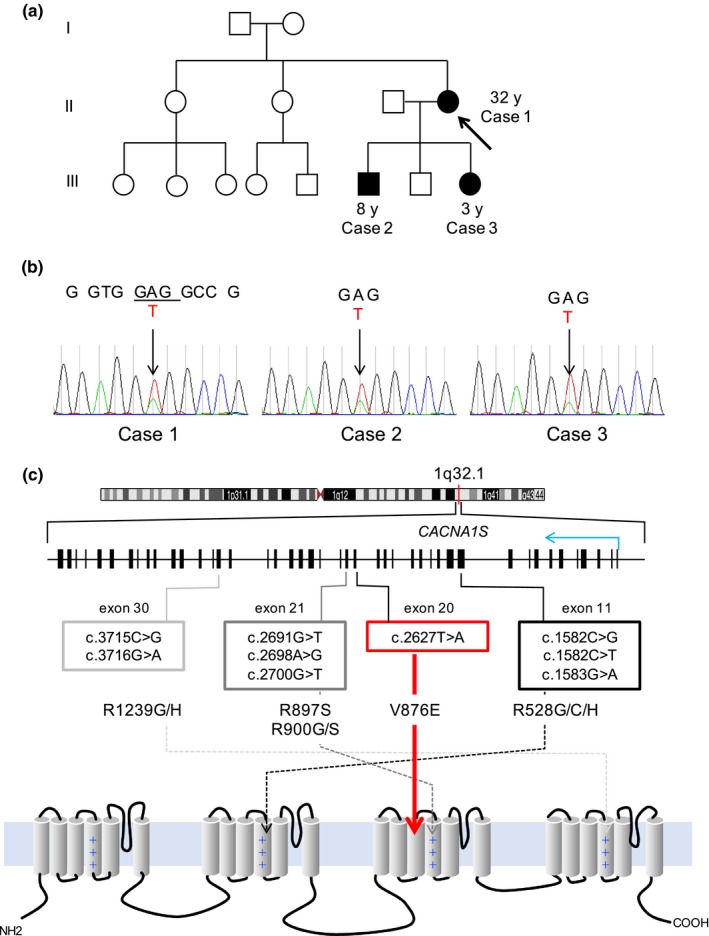
Summary of the family and mutations in *CACNA1S*. (a) The pedigree chart for the three cases. Case 1 (arrow) was the proband of hypokalemic periodic paralysis (HypoPP) in this family. This report also presents the clinical features of her son (8 years of age) and daughter (2 years of age). (b) Sequencing chromatograms of Cases 1–3. Note that these patients showed the heterozygous mutation of T>A at the GTG codon (Val876) in the coding region of *CACNA1S*. (c) The *CACNA1S* locus on chromosome 1 and the mutation map on the gene (middle) and Ca_v_1.1 protein. Note that *CACNA1S* is located at the minus strand of human genome, and that the first exon is shown at the right end. The blue arrow in the middle panel indicates the direction of transcription. The annotated boxes denote previously reported mutations (gray to black) and that identified in this family (red, V876E). The bottom panel depicts the transmembrane domains (barrels) and the loop structure (black curves) of Ca_v_1.1. Plus (+) symbolizes the positive charges in segment 4 of the calcium channel

*Case 1:* A 32‐year‐old Japanese woman was the proband of this family (Figure 1a). When she presented with the first attack at 6 years of age, the potassium level was decreased to 1.9 mEq/L in serum. She thus received the diagnosis of idiopathic HypoPP because no family members were reportedly affected at that time. Each paralytic symptom usually ceased in 4–5 hr, and the frequency of paralysis was once in a few months. Her motor, language, and social development were all normal. She experienced daily recurrence of paralysis at 13 years of age. The exacerbation was thought to be associated with the insufficient intake of potassium and the high‐carbohydrate diet. Laboratory tests detected an increase in the immunoreactive insulin during the paralytic symptoms, and the 75‐g oral glucose tolerance analysis supported evidence for the excessive insulin secretion in serum. Peroral administration of voglibose and acetazolamide prevented the paralytic attacks thereafter. While acetazolamide was discontinued during her pregnancy at 25 years of age, paralytic symptoms reactivated. However, she gave birth to three children without perinatal complications or further progression of symptoms. She presently takes voglibose, oral potassium supplementation (36 mmol/day), and acetazolamide. Mild attacks of paralysis occur at a frequency of a few times in a week.

*Case 2* is the first child of *Case 1* (Figure 1a). He was born at 37 weeks of gestation without asphyxia. His birth weight (2,635 g), height (47.0 cm), and head circumference (33.5 cm) were appropriate for date. His psychomotor development was normal in infancy. When he had gastroenteritis at 4 years of age, he was unable to stand or walk unaided. The onset of HypoPP was suspected because the chemistry serum revealed the decrease in potassium to 2.9 mEq/L. He also developed the second attack of weakness when he had the respiratory infection 1 month after the first episode. In both events, the muscle weakness spontaneously ameliorated within a day. He was referred to our department when he developed the third paralytic episode on influenza infection at 6 years of age. Vital signs were unremarkable, including blood pressure (108/64 mmHg). The electrocardiogram showed ST‐segment depression and U waves. Blood tests showed the decreased level of potassium to 1.9 mmol/L and increased levels of creatine kinase (CK) to 502 U/L (normal range: 52–248), plasma renin activity (PRA) to 12 ng ml^−1^ hr^−1^ (normal range: 0.8–2.0), and aldosterone to 483 pg/mL (normal range: 55–142). The potassium/creatinine ratio (10.0 mEq/g Cr, cutoff line <22; Lin et al., 2004) was low. The paralysis was improved by intravenous infusion of potassium within 2 hr. However, PRA (4.1 ng ml^−1^ hr^−1^) was still higher than the normal range even after the recovery of muscle strength. Aldosterone returned to the normal range at this age (91.8 mmol/L). He showed no hypertension during the study period. Oral glucose tolerance test excluded the abnormal secretion of insulin. Since he started the oral potassium supplementation (48 mmol/day), he had only fatigues without paralytic symptoms on respiratory infections or after exercise. Chemistry serum showed that CK was constantly high (310–631 U/L) in the follow‐up studies. At presently 8 years of age, his growth and mental development are normal. He walks and jumps normally, but he cannot run fast. The constantly high CK level and his symptoms suggested the possibility of HypoPP‐related myopathy. He attends regular elementary school without intellectual handicaps.

*Case 3.* This 36‐month‐old girl is the third child of *Case 1* (Figure 1a). She was born at 39 weeks of gestation after uneventful pregnancy and delivery. Her psychomotor development was normal in infancy. She first presented with the paralytic attack when she developed pharyngitis at 2 years and 4 months of age. When she visited our hospital on the first day of this event, we determined that the serum potassium level was decreased to 2.9 mmol/L. Serum CK was elevated to 2,512 U/L. Because the paralytic symptom disappeared within 1 day after the onset, PRA and the aldosterone concentration were not tested during this event. However, we found that PRA (24 ng ml^−1^ hr^−1^) and aldosterone (151 pg/mL) levels were both higher than the normal range 1 day after the paralytic event. She then started taking oral potassium only at the time of febrile illness. She had no recurrence of paralytic symptoms for 9 months. Follow‐up studies of chemistry serum revealed that CK was continuously high (505–626 U/L). At present, her physical growth, motor, and cognitive development are normal.

## 3 GENETIC ANALYSIS

Targeted, next‐generation amplicon sequencing with Ion PGM (Thermo Fisher Scientific) revealed that *Case 1* carried a heterozygous pathogenic variant (NM_000069.2: c.2627T>A: p.V876E) in *CACNA1S*, but not in *SCN4A, KCNJ2, or KCNJ5*. The Sanger method verified that *Cases 2* and *3* also had the same mutation heterozygously (Figure 1b). We have not tested whether the healthy sibling, the second child of the proband, had this mutation. We found that two non‐Japanese families were previously reported to carry the identical mutation to these cases (Figure 1c; Ke et al., 2009; Yang et al., 2015).

## 4 DISCUSSION

In this report, we have identified the first Japanese family harboring the p.Val876Glu mutation in *CACNA1S.* Because only two families with HypoPP have been reported to carry this mutation, our data further strengthened the uniqueness of p.Val876Glu regarding its structural location in Ca_v_1.1 and the clinical phenotype associated with the mutation (Ke et al., 2009; Yang et al., 2015).

Most of HypoPP‐associated mutations in Nav1.4 and Ca_v_1.1 have been reported to occur at positively charged arginine called “gating charges” in segment 4 (S4) of the voltage sensor (Matthews et al., 2009). Loss of the positive net charge in S4 was therefore considered important for the generation of gating pore current (Matthews et al., 2009). On the other hand, p.Val876Glu in *CACNA1S* is neither located in S4 nor leads to a loss of the positive charge. Thus, the pathogenic impact of this mutation remained inconclusive when it was originally reported (Ke et al., 2009). Nevertheless, biophysical evidence showed that Ca_v_1.1 with p.Val876Glu revealed monovalent cation leak inward currents at resting potential most likely through the gating pore, resulting in contributing a pathogenic condition that plausibly explained the severe HypoPP symptoms (Fuster et al., 2017). The experimental data supported a concept that the functional conformation of the voltage sensor is not only sustained by the positively charged amino acid residues in the S4 segment but also by an external amino acid residue (Val876) in other segments of the voltage sensor.

Clinically, the patients with p.Val876Glu mutation showed an earlier onset of a paralytic attack. To date, five in two other families have been reported to carry the p.Val876Glu mutation in *CACNA1S* (Ke et al., 2009; Yang et al., 2015). These patients showed the periodic symptoms of paralysis at earlier ages (5.2 ± 3.6 years) compared to those with other mutations (p.Arg528His [14 ± 5 years] and p.Arg1239His [7 ± 4 years]; Miller et al., 2004). Consistent with these results, our patients developed the symptoms of HypoPP at 2–6 years of age, which were younger than the typical age of onset. Detailed mechanisms of the variable phenotypes in this family or the age of onsets remain unknown. However, considering the unique presentation of excessive insulin secretion in Case 1 and the increased levels of renin activity in Cases 2 and 3, *CACNA1S* may play distinct roles in regulating the cellular excitability at different ages in childhood.

Case 1 showed excessive secretion of insulin on the physiological condition and on high‐carbohydrate diets. We also found elevated levels of PRA in Cases 2 and 3. To date, no reports have shown endocrine dysfunctions, elevated PRA, or aldosterone in patients with HypoPP. We confirmed that one patient with HypoPP (p.Val876Glu) had normal PRA and aldosterone (Yang et al., 2015). However, endocrine profiles were not reported for the other case with a p.Val876Glu mutation. It is accepted that another voltage‐gated calcium channel Ca_v_1.3 regulate glucose‐induced insulin secretion from the pancreatic beta cells (Reinbothe et al., 2013). By contrast, the functional role of Ca_v_1.1 in pancreas and kidney has never been investigated thus far, and the endocrine phenotype of HypoPP has never been described elsewhere. We therefore need to await next reports to confirm that mutations in *CACNA1S* are directly or indirectly linked to the non‐neuromuscular phenotypes of HypoPP.

We also found that serum CK levels were constantly high in Cases 2 and 3. It is well known that patients with HypoPP may show transient increase in CK on episodic paralysis (Hirano et al., 2011; Kawamura, Ikeda, Tomita, Watanabe, & Seki, 2004). However, sustained elevation of CK is an uncommon finding in affected patients. Through the literature search, we found one report presenting such a case with sustained elevation of CK (Neame, Wright, & Chandrasekaran, 2017). This patient was reported to have a mutation in *CACNA1S*; however, the genotype was not disclosed in this report. Although the precise mechanisms remained unexplained, we considered this finding an asymptomatic sign of myopathy. In fact, recent studies showed the frequent comorbidity of myopathy with HypoPP (Sternberg et al., 2001). One could hypothesize that the high CK and renin activities in this case might be directly or indirectly linked to the phenotypes of children with the p.Val876Glu mutation in *CACNA1S*.

This family showed unusual findings of hyperinsulinemia, elevated PRA, and persisting hyper‐CK levels, but none of these findings was commonly observed among mother and her two children. Thus, we cannot safely conclude that p.Val876Glu is associated with these findings even in this family. Subsequent studies on nonmuscular phenotypes will provide further insight into the genotype–phenotype correlations in pediatric‐onset HypoPP.

## ETHICS STATEMENTS

This study was approved by the Institutional Review Board at Kyushu University (No. 461‐04) and Osaka University. Written informed consent was obtained from Case 1 and her spouse (parents of Cases 2 and 3) prior to the genetic analysis for presenting the clinical data in this manuscript.

## CONFLICT OF INTEREST

None to disclose.

## Data Availability

The data that support the findings of this study are available on request from the corresponding author. The data are not publicly available due to privacy or ethical restrictions.
